# Management of Hirschsprung’s Disease: A Survey with Brazilian Pediatric Surgeons

**DOI:** 10.3390/children11111405

**Published:** 2024-11-20

**Authors:** Cesar Saul Quevedo Penaloza, Alana Carnevale Barreto, Erika Veruska Paiva Ortolan, Augusto Zani, Pedro Luiz Toledo de Arruda Lourenção

**Affiliations:** 1 Botucatu Medical School, São Paulo State University (UNESP), Botucatu 18618-970, São Paulo, Brazil; cesar.quevedo@hc.fm.usp.br (C.S.Q.P.); alana.barreto@unesp.br (A.C.B.); erika.ortolan@unesp.br (E.V.P.O.); 2 Department of Surgery, Division of General and Thoracic Surgery, The Hospital for Sick Children, University of Toronto, Toronto, ON M5G 1E8, Canada; augusto.zani@sickkids.ca

**Keywords:** Hirschsprung disease, congenital megacolon, colonic aganglionosis, Soave, Duhamel, transanal

## Abstract

Background: Hirschsprung’s disease (HD) is a congenital malformation of the enteric nervous system clinically manifested by intestinal obstruction in the neonatal period or severe constipation in childhood. Several surveys on HD have been conducted to evaluate experiences in its management around the world. For the first time in Brazil, we analyze and report the management patterns of HD among pediatric surgeons in Brazil. Methods: A validated questionnaire was disseminated in print at the Congress of Pediatric Surgery in São Paulo-Brazil, and an online version was sent to all the active members of the Brazilian and Paulista Institute of Pediatric Surgery. Results: In total, 361 pediatric surgeons answered the survey. Of these, 329 completed all questions (response rate: 91%). Most Brazilian services treat fewer than 10 cases of HD annually. The preferred diagnostic method was rectal biopsy. For newborns (NBs) and infants, open biopsy was the most commonly used technique. For NBs with HD clinically stable 50% of specialists chose immediate surgery. In NBs and infants with classic HD, the Soave technique (69%) is the most common surgical intervention, and the transanal route (80%) is the preferred surgical approach. In children over 3 years of age with classic HD, the most-used technique is the Duhamel method (54%), with the open approach being the most common (52%). Conclusions: Our study in Brazil found that HD patient management aligns with scientific evidence and international guidelines.

## 1. Introduction

Intestinal dysganglionoses encompass a range of disorders related to the enteric nervous system, one of which is Hirschsprung disease (HD), also referred to as congenital megacolon, which is characterized by the absence of ganglion cells in the distal colon, leading to functional obstruction [1,2].

After close to 150 years since its first description by Harald Hirschsprung, a Danish pediatrician, this condition has been extensively researched. Issues concerning HD’s etiology [3], diagnosis, and treatment continue to be a subject of research and discussion in the literature for better managing children with this condition [4]. However, the deficiency of randomized clinical trials in pediatric surgery and HD is predominantly due to the rarity of such cases and ethical issues related to child studies [5]. In the case of a scarcity of such trials, surveys have emerged as an alternative approach for the study of low-frequency conditions such as HD. Such surveys provide quite useful information related to clinical practice comparisons, evidence-based reviews, and fresh research perspectives. However, it is important that the methodology be carefully executed and well analyzed in order to produce meaningful results [6,7].

Several surveys of HD have been conducted to assess experiences in its management. In 1979, the American Academy of Pediatrics published the first survey [8], while the American Pediatric Surgical Association (APSA) conducted a new survey in 2009, observing changes in surgical treatment [6]. In the United Kingdom, opinion polls were conducted in 1998 and 2010, showing changes towards less invasive surgeries. In Japan, three surveys between 1978 [9] and 2002 [10] demonstrated changes with the introduction of laparoscopy and transanal surgery [6]. Surveys by the European Association of Pediatric Surgeons (EUPSA) [11] and the Australian and New Zealand Association of Pediatric Surgeons (ANZAPS) [12] also reported wide variation in diagnostic and therapeutic approaches between different centers. In 2021, an online survey was conducted in Latin America, showing the current practice of rectal biopsies for the diagnosis of HD [13]. The objective of our study was to analyze and present the management patterns of Hirschsprung disease among pediatric surgeons in Brazil.

## 2. Materials and Methods

### 2.1. Study Design and Scenario

This is a survey study. This type of research is a quantitative research method, defined as a way of analyzing data and information based on the characteristics and opinions of groups of individuals.

### 2.2. Ethical Aspects of This Study and Registry

The Internal Review Board (IRB) of the Botucatu Medical School, UNESP, São Paulo, Brazil, gave its approval to this research, which has registration number CAAE 34480920.2.0000.5411. Every participant completed an informed consent form after being previously briefed on this study’s objectives (Supplementary Materials S1).

### 2.3. Eligibility Criteria

Our survey was conducted in Brazil with physicians working in the area of pediatric surgery, including those who have a medical residency or specialist degree in pediatric surgery and physicians who are residents in pediatric surgery. In 2022, the year in which the application of the questionnaire was completed, the Brazilian Association of Pediatric Surgery (BAPS) had 454 active members, including pediatric surgeons and pediatric surgery fellows [14].

### 2.4. Preparation of the Questionnaire

We obtained authorization, from the author Zani, to translate and adapt into Portuguese his tool used during the EUPSA survey (2017) (Supplementary Materials S2) [11]. The translated questionnaire (Supplementary Materials S3) underwent a pre-test with 15 volunteer Brazilian pediatric surgeons to ensure comprehension and refinement of the language (Supplementary Materials S4) [7,15]. Following the discussion of pre-test feedback, we finalized the questionnaire for the collection phase (Supplementary Materials S5) [16,17]. The questionnaire covered aspects of HD, including diagnosis, surgery, and specific patient cases like trisomy 21 and total colonic aganglionosis.

### 2.5. Interventions

Data collection occurred in two stages. The first was conducted between August 2021 and February 2022; we contacted members of the BAPS through email, WhatsApp, and Instagram to fill out an online questionnaire using the platform Survey Monkey, which guarantees respondent confidentiality. The second stage occurred in March 2022 during the XIII Congress of Pediatric Surgery, where a printed questionnaire was distributed to BAPS members who had not participated online. Responses were collected anonymously at the exit of the main hall of the congress, ensuring confidentiality.

## 3. Results

### 3.1. Participants

In total, 361 pediatric surgeons answered the survey, 274 online (76%) and 87 in print (24%). Of these, 329 completed all questions, resulting in a 91% response rate, with 253 (77%) answering online and 76 (23%) using printed forms. The average duration for completing the questionnaire was seven minutes. Participants were asked to indicate their position and the location where they work. Out of the 329 participants, 76 identified as heads of service (23%), 201 as permanent staff or consultants (61%), and 52 as fellows (16%). Regarding the reported locations of practice, 57% were in the Southeast region of the country, 17% in the Northeast, 15% in the South, 6% in the North, and 5% in the Midwest.

### 3.2. Center

In total, 272 respondents (83%) indicated that they work at centers that handle fewer than 10 HD cases per year, while 46 respondents (14%) are at centers that manage between 10 and 20 HD cases each year, and 11 respondents (3%) are at centers treating over 20 HD cases annually.

### 3.3. Diagnosis

**Figure 1:**
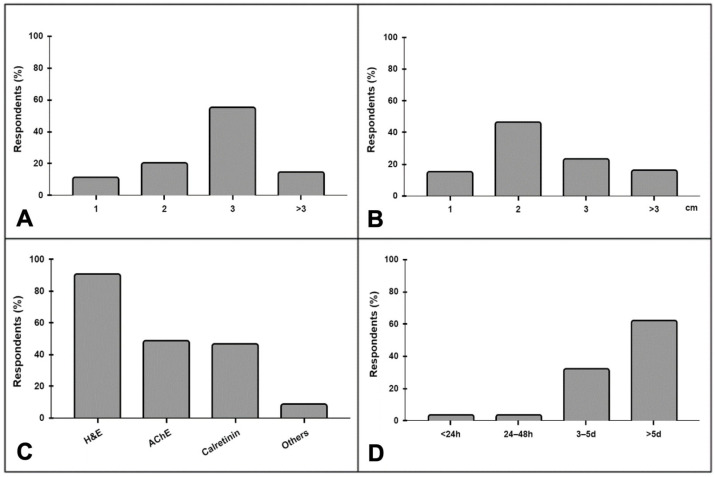
Variations in rectal biopsies used for the diagnosis of HD: (**A**) number of specimens routinely taken; (**B**) location of the most distal biopsies from the dentate line; (**C**) most used histological and immunohistochemical methods; (**D**) time to the pathology report.

During the assessment of patients with suspected HD, 95% of respondents request rectal biopsy, 98% use contrast enema, 21% perform anorectal manometry, and 4% use other tests. In neonates and infants, rectal biopsies are obtained via open full-thickness by 84% of respondents, using the suction technique by 11%, and 5% use other methods, mostly by punch. In children over 3 years old, rectal biopsy is obtained via open full-thickness by 86%, while 7% use the suction technique, and 6% use other methods. Further, 55% of respondents typically collect three specimens for biopsy, while 20% collect two, 14% collect more than three, and 11% take only one (Figure 1A). Most distal biopsies are performed at 2 cm from the dentate line, by 46% of the respondents, while 23% take it at 3 cm from the dentate line, 16% at more than 3 cm, and 15% at 1 cm (Figure 1B). Regarding the histological and immunohistochemical methods for diagnosing HD, the most used are hematoxylin/eosin (90%), acetylcholinesterase (48%), and calretinin staining (46%). Other markers, such as S100, enolase and C-kit, are used less often (8%) (Figure 1C). In addition, 62% of surgeons indicated that it takes more than 5 days to receive biopsy reports, while it takes 3 to 5 days for 32%, 24 to 48 h for 3%, and less than 24 h for another 3%. (Figure 1D). Most surgeons (95%) acknowledge the existence of other intestinal dysganglionoses, including hypoganglionosis (83%), intestinal neuronal dysplasia type B (77%), ultrashort segment HD (68%), and desmosis coli (8%).

### 3.4. Surgery

When a neonate is diagnosed with confirmed HD, 51% of respondents indicate that the next step in management is to perform an immediate pull-through (PT) procedure. Conversely, 49% of respondents choose to postpone the PT for the neonatal period to 6 months of age or when the infant weighs more than 5 kg. While awaiting surgery, 73% of respondents keep the bowel decompressed using home rectal irrigations, while 25% utilize home rectal dilation/stimulation, and 30% opt to create a stoma. If a stoma is performed, 50% place it at the level of the colon dilation transition, 23% locate it in the sigmoid colon, 9% in the right transverse colon, 1% in the ileum, and 17% in other sites.

**Figure 2:**
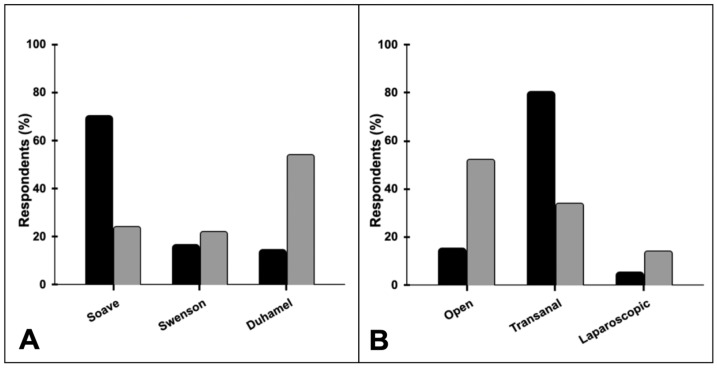
Responses about preferred definitive surgery: (**A**) type of PT; (**B**) approach used for the PT. Newborns or infants (black) and children over 3 years old (gray).

In the case of typical recto-sigmoid HD, the preferred type of PT in newborns and infants is the Soave or De La Torre-Mondragon approach transanal technique (70%), followed by the Swenson PT abdominoperineal or transanal technique (16%) and Duhamel PT (14%) (Figure 2A). For these patients, 80% of respondents performed the PT by the transanal technique, 15% by the open technique, and 5% by laparoscopy (Figure 2B). In children over 3 years old with classic HD, the preferred approach is the Duhamel (54%), followed by Soave or De La Torre (24%) and Swenson (22%) (Figure 2A). In this age group, the PT is performed by the open technique by 52%, followed by the transanal (34%) and the laparoscopic (14%) (Figure 2B). PT is planned to happen in the neonatal period by 22% of respondents, between 1 and 6 months of age by 48%, between 6 months and 1 year by 23%, and older than a year by 7%.

If symptoms persist after a “successful” PT, 60% of respondents opt for a conservative approach with enemas and laxatives, 11% would redo the PT, 10% opt for botulinum toxin injection, and 8% perform a posterior myectomy. In patients with trisomy 21, the majority of surgeons (95%) do not alter their surgical strategy. Those interviewed changed their planning to research comorbidities and associated syndromes.

### 3.5. Total Colonic Aganglionosis

In patients with a diagnosis of Total Colonic Aganglionosis (TCA), 5% of respondents perform the PT when the patient is older than 4 years, 15% between 2 and 4 years, 34% between 1 and 2 years, 28% between 6 months and 1 year, 13% between 1 and 6 months of age, and only 5% during the neonatal period. If a stoma is necessary, 49% of respondents said they always place that in the ileum, 28% said they did so based on intraoperative biopsies, 21% said they did so based on the macroscopic impression made during surgery, and 2% said they did so based on radiological data. The Duhamel method is the most popular physical therapy for patients with TCA (67%), followed by Swenson (17%), Soave (7%), and others (9%). In the context of performing a “J” ileal pouch in patients with TCA, a majority of specialists (55%) reported that they do not perform this option.

## 4. Discussion

This first survey of practice among pediatric surgeons in Brazil revealed a lack of consensus in most aspects of HD management. This study had a significantly larger number of respondents compared to previous HD research [8,9,10,11,12]. The distribution of respondents closely matched the demographic distribution of pediatric surgeons in Brazil according to the 2023 medical demographics report [18], highlighting the unequal availability of resources in health services across the country. The majority of respondents were permanent staff or consultants practicing in hospitals that treat fewer than 10 HD cases per year.

In this survey, one of the most striking outcomes is the variability in obtaining the diagnosis of HD. The most commonly performed tests in diagnostic investigation were contrast enema (98%) and biopsy (95%). Only 20% of respondents reported using anorectal manometry. It is less commonly used, but it has higher sensitivity and specificity values than barium enema [4]. It is well-established as a diagnostic screening method, but it is more expensive and less available. It is important to highlight that 95% of respondents are diagnosing with rectal biopsies, which is considered the gold-standard examination essential for diagnosing HD [19,20,21,22,23], but there is inconsistency in the methods used for obtaining, processing, and interpreting these biopsies.

In the context of rectal biopsies, the open surgical technique was found to be the most commonly employed method, irrespective of the patient’s age. The distinction affects the requirement for sedation or anesthesia in the open technique in addition to being purely technical in terms of specimen collection. Furthermore, there is a great deal of variation in the quantity of specimens gathered as well as the precise sites of biopsies.

Only about 10% of practitioners use suction forceps for biopsies. This is different from Europe [11] and Oceania [12], where suction forceps biopsies are the most commonly used technique. Suction forceps biopsies are minimally invasive and have low complication rates, with similar conclusive results to open surgical techniques [4]. The limited use of this method in Brazil may be due to the lack of suction forceps. None of the forceps’s types are regularly available in our country and they are not registered with the National Health Surveillance Agency. In Latin American countries, about 14% of respondents in a recent survey reported using unregistered, hand-made forceps for suction biopsy in histological diagnosis [13].

To perform rectal biopsies, the majority of respondents opt to collect two to three specimens, with most distal fragments obtained 2 to 3 cm above the pectinate line. These are similar to those obtained in recent surveys conducted in Europe [11], Oceania [12], and Latin America [13]. The histopathological analysis methods commonly used in our country and the time to obtain the biopsy result are similar to international practices and guidelines already published [11,12,13,23].

Concerning other diseases of the enteric nervous system, which represent differential diagnoses for HD, most respondents believe in the existence of Intestinal Neuronal Dysplasia Type B, hypoganglionosis, and the ultra-short form of HD. However, only a minority reported seeing and diagnosing cases of these diseases [24,25,26]. Cases of these pathologies were often found in isolation, without association with HD. Similar findings were reported by Zani in a survey of European pediatric surgeons [11].

Concerning therapeutic approaches, when diagnosing HD in a stable newborn, almost half of the respondents (49%) reported opting to postpone surgery for between 1 and 6 months, preferably maintaining the patient with rectal irrigations at home [26]. Performing primary PT, without a stoma, and keeping the colon decompressed with rectal irrigations is the preferred trend reported in the literature. In past surveys, the majority of respondents from Europe [11] and Oceania [12] indicated a preference for surgical treatment during the neonatal period. However, there is currently no scientifically supported agreement on the most suitable approach. Therefore, this decision should consider the individual clinical conditions of each patient, as well as the expertise and technical training of each medical facility. In 2022, the publication of a systematic review with meta-analysis demonstrated a tendency towards a worsening in the functional prognosis of children undergoing primary endoanal PT before 45 days of life [27,28]. When intestinal derivation was necessary, most respondents reported performing stomas preferably just above the transition region between the aganglionic segment and the dilated region, a practice widely used in the literature [29].

Among all pediatric surgical diseases, HD continues to be one of the most complex and contentious surgical conditions. Various surgical techniques for treating HD have evolved from methods by Orvar Swenson (1948), Bernard Duhamel (1956), and Franco Soave (1963) [17]. Our survey shows that the most common approach for newborns and infants is PT via transanal using the Soave technique [30], similar to pediatric surgeons in Europe and Oceania [11,12]. Endoanal techniques are currently preferred for performance [31]. In a systematic review and meta-analysis, it was found that transanal PT showed a greater tendency for enterocolitis and anastomotic stenosis compared to the Duhamel technique [28]. However, they had a lower tendency for constipation, incontinence, fecal loss, and anastomotic dehiscence. Among transanal techniques, there is no consensus on the most appropriate technique in terms of complications and long-term follow-up. A comparative study found that the Swenson transanal PT technique had less blood loss, shorter surgical time, and lower complication rates compared to the technique proposed by De La Torre and Ortega-Salgado [31]. For children older than 3 years, our professionals primarily employed open Duhamel surgery (54%); in contrast, laparoscopic-assisted mobilization was the least frequently utilized technique; however, it presents significant advantages, particularly in relation to the markedly shortened transanal procedure. This innovative approach is increasingly recognized as a viable alternative in contemporary practices.

A delayed diagnosis of HD is associated with significant dilation and thickening of the colon. This dilation can lead to technical challenges, such as difficulties in performing video laparoscopic assistance for colon dissection and endoanal resection, necessitating greater traction force from detractors and generally longer surgical time. Prolonged stretching of the anal sphincter subsequently may result in long-term deficits in continence mechanisms. Due to these concerns, the use of exclusive endoanal techniques in older children has been called into question [27]. Our survey reflects this trend, as the Duhamel technique is the preferred approach for children over 3 years old, performed via open surgery.

The majority of responders said they preferred conservative treatment with laxatives and/or intestinal lavages when obstructive symptoms persisted following an adequate colonic resection. Notably, the utilization of botulinum toxin has been increasingly advocated in this context, supported by recent guidelines and showing promising outcomes [32,33,34]. However, due to its recent introduction as a therapeutic option, this approach is not yet widely adopted in our country.

There is no consensus on the clinical and surgical management of children with total colonic aganglionosis. The timing and techniques for surgical treatment vary, and there is a lack of consensus in responses obtained from surveys. Recent proposals suggest using clinical and laboratory criteria to determine the best timing for surgical treatment and intestinal transit reconstruction [35]. The choice of surgical technique currently depends on the surgeon’s training and individual patient factors.

Utilizing a structured questionnaire and an online completion tool can mitigate biases in opinion polls. Keep in mind that this study is purely descriptive and is based on expert opinions. Nonetheless, in situations where conducting randomized and multicenter clinical studies is challenging, such as in the cases of rare diseases, surgical conditions, and pediatric patients, this type of study can offer valuable insights for clinical practice [36,37]. It can shed light on commonly utilized procedures, facilitate comparisons between different contexts, and potentially inform the establishment of local guidelines.

## 5. Conclusions

This study concludes that there are notable differences in HD diagnosis and treatment in Brazil. This is seen in the variety of rectal biopsies obtained, the kind of surgery performed, and the general way HD patients are managed. Open biopsies are still more frequently performed. In neonates and infants, the most frequent surgical method employed for PT is the transanal Soave. For children older than 3 years, our specialists generally use open Duhamel surgery. Approaches on how to treat patients with TCA are very different. Based on the information, a national guideline for diagnosis and treatment should be developed that aligns with scientific evidence and international literature.

## Data Availability

Data are contained within the article and Supplementary Materials.

## Supplementary Materials

The following supporting information can be downloaded at: https://www.mdpi.com/article/10.3390/children11111405/s1, Supplementary Material S1: Internal Review Board of the Botucatu Medical School, UNESP, São Paulo, Brazil; Supplementary Material S2: HD international survey, EUPSA (2017); Supplementary Material S3: EUPSA survey (2017) adapted and translated into Portuguese; Supplementary Material S4: Pre-Test: “Brazilian Questionnaire on Hirschsprung’s Disease”; Supplementary Material S5: Final version: “Brazilian Questionnaire on Hirschsprung’s Disease”.
