# Supplementary material for: Management of Hirschsprung’s Disease: A Survey with Brazilian Pediatric Surgeons
Source: Children (Basel). 2024 Nov 20;11(11):1405. doi: 10.3390/children11111405 (PMC11592611; doi:10.3390/children11111405)
Supplement: Supplementary file 1 [file children-11-01405-s001.zip › children-3251630-supplementary.pdf]

Internal Review Board of the Botucatu Medical School, UNESP, São Paulo, Brazil

UNESP -FACULDADE DE MEDICINA DE BOTUCATU

PARECER CONSUBSTANCIADO DO CEP

DADOS DO PROJETO DE PESQUISA

Título da Pesquisa: Manejo diagnóstico e terapêutico da Doença de Hirschsprung: um levantamento com cirurgiões pediátricos do Brasil

Pesquisador: Cesar Saul Quevedo Penaloza

Área Temática:

Versão: 2

CAAE: 34480920.2.0000.5411

Instituição Proponente: Departamento de Cirurgia e Ortopedia

Patrocinador Principal: Financiamento Próprio

DADOS DO PARECER

Número do Parecer: 4.287.374

Apresentação do Projeto:

Trata-se de um estudo de pesquisa de opinião (tipo survey), com cirurgiões pediátricos atuantes no país.

O presente estudo está orientado a falar sobre a experiência em manejo de doença de Hirschsprung pelos cirurgiões pediátricos do Brasil e será realizado no ano 2020-2021, estudando variação no diagnóstico e tratamento da doença, pelos profissionais nos distintos estados do país.

O projeto informa a inclusão de 827 participantes.

Objetivo da Pesquisa:

Realizar um levantamento das condutas diagnósticas e terapêuticas, realizadas por cirurgiões pediátricos,em pacientes com Doença de Hirschsprung (DH), permitindo o conhecimento das práticas mais utilizadas no Brasil.

Avaliação dos Riscos e Benefícios:

Os riscos são mínimos, desde que mantida a confidencialidade.

Benefícios:Proporcionará conhecimento sob o manejo dos cirurgiões pediátricos do Brasil da Doença de Hirschsprung.

Comentários e Considerações sobre a Pesquisa:

Trata-se de um estudo relevante e factível.

O estudo acontecerá em 2 fases: 1) pré-teste para validação de matéria;

2)aplicação de um questionário: Serão enviados convites por correspondência eletrônica para cirurgiões pediátricos do Brasil. Estes endereços serão obtidos por solicitação à Associação Brasileira de Cirurgia Pediátrica e demais associações estaduais de Cirurgia Pediátrica. O preenchimento do questionário será realizado através do software Survey Monkey, uma plataforma de pesquisa online, que garante a confidencialidade da identificação dos respondentes e também está planejada a aplicação dos questionários no Congresso Brasileiro de Cirurgia Pediátrica de 2021 e outros congressos da especialidade.

Considerações sobre os Termos de apresentação obrigatória:
Apresentam-se anuências institucionais e TCLE aos participantes.

Recomendações:
apresentar relatório final de atividades após finalização da pesquisa.

Conclusões ou Pendências e Lista de Inadequações:
Após análise em REUNIÃO ORDINÁRIA, o Colegiado deliberou APROVADO o Projeto de Pesquisa apresentado.

Considerações Finais a critério do CEP:
Conforme deliberação do Colegiado, em REUNIÃO ORDINÁRIA do Comitê de Ética em Pesquisa FMB/UNESP, realizada em 08/09/2020, o Projeto de Pesquisa apresentado encontra-se APROVADO.
O Pesquisador deverá enviar RELATÓRIO FINAL DE ATIVIDADES após finalização da pesquisa, via notificação junto à PB.
O projeto de pesquisa deverá ser iniciado após aprovação do CEP.

Atenciosamente, Comitê de Ética em Pesquisa FMB/UNESP

Este parecer foi elaborado baseado nos documentos abaixo relacionados:

Tipo Documento	Arquivo	Postagem	Autor	Situação
Informações Básicas do Projeto	PB_INFORMAÇÕES_BÁSICAS_DO_PROJETO_1588344.pdf	19/08/2020 12:47:36		Aceito

Outros	Oficio_resposta.doc	19/08/2020 12:47:11	Pedro Luiz Toledo de Arruda Lourenção	Aceito
TCLE / Termos de Assentimento / Justificativa de Ausência	TCLE_participantes_survey.doc	19/08/2020 12:45:38	Pedro Luiz Toledo de Arruda Lourenção	Aceito
TCLE / Termos de Assentimento / Justificativa de Ausência	TCLE_resumido.doc	19/08/2020 12:45:19	Pedro Luiz Toledo de Arruda Lourenção	Aceito
TCLE / Termos de Assentimento / Justificativa de Ausência	TCLE_pre_teste.doc	19/08/2020 12:44:41	Pedro Luiz Toledo de Arruda Lourenção	Aceito
Outros	termodeanuenciainstitucional.pdf	03/07/2020 16:15:06	Cesar Saul Quevedo Penaloza	Aceito
Folha de Rosto	folhaderostoassinada.pdf	03/07/2020 16:14:05	Cesar Saul Quevedo Penaloza	Aceito
Cronograma	cronograma.pdf	02/07/2020 21:16:55	Cesar Saul Quevedo Penaloza	Aceito
Projeto Detalhado / Brochura Investigador	projeto.pdf	02/07/2020 21:16:25	Cesar Saul Quevedo Penaloza	Aceito

Situação do Parecer:
Aprovado

Necessita Apreciação da CONEP:
Não

BOTUCATU, 19 de Setembro de 2020

Assinado por:
SILVANA ANDREA MOLINA LIMA
(Coordenador(a))

Endereço: Chácara Butignolli , s/n
Bairro: Rubião Junior **CEP:** 18.618-970
UF: SP **Município:** BOTUCATU
Telefone: (14)3880-1609 **E-mail:** cep@fmb.unesp.br

Supplementary Material S2

Penaloza et al. Management of Hirschsprung's Disease: A Survey with Brazilian Pediatric Surgeons

EUPSA survey (2017)

Hirschsprung's disease International Survey

Head of Department / Permanent Staff or Consultant / Trainee (please circle as appropriate)

Hospital :...Country.....................................

How many cases of Hirschsprung's disease (HD) does your centre manage a year?
☐ <10 ☐ 10-20 ☐ >20

Diagnosis

In the work-up of patients with suspected HD, which tests do you perform?

☐ Rectal biopsy ☐ Contrast enema ☐ Anorectal manometry

In case of rectal biopsy, what is your approach?

☐ Suction biopsy ☐ Open biopsy

In case of rectal suction biopsy,

how many biopsy specimens do you usually take? ☐ 1 ☐ 2 ☐ 3 ☐ >3

how many cm from the dentate line is your most distal biopsy? ☐ 1 ☐ 2 ☐ 3 ☐ >3

For rectal biopsies, which staining does your pathologist use? *(Can tick more than one)*

☐ Hematoxylin/eosin ☐ Acetylcholinesterase

☐ Calretinin ☐ Other *(please specify)* ....

How long do you have to wait to get a rectal biopsy report?

☐ <24h ☐ 24-48h ☐ 3-5 days ☐ >5 days

Which of the following entities do you recognise?

☐ Intestinal neuronal dysplasia ☐ Hypoganglionosis ☐ Ultra-short segment HD

☐ Desmosis coli ☐ none of the above ☐ Other *(please specify)* ....

Surgery

In a neonate with confirmed HD, what is your immediate management?

☐ Operation at diagnosis ☐ Delayed operation

If you delay surgery, when do you plan the pull-through?

....

Continues ⇨

Whilst waiting for surgery, how do you keep the bowel decompressed?

☐ Rectal irrigations at home ☐ Rectal dilatation/stimulations at home

☐ Primary pull-through ☐ Stoma ☐ Other *(please specify)* ....

If you create a stoma in a patient with typical recto-sigmoid HD, where do you site it?

☐ Sigmoid colon ☐ Right transverse colon ☐ Ileum ☐ Levelling stoma

At what age do you plan the pull-through?

☐ Neonatal period ☐ 1-6 months ☐ 6-12 months ☐ > 12 months ☐ older

Which type of pull-through do you favour? ☐ Soave ☐ Swenson ☐ Duhamel

Which approach you use to perform the pull-through?

☐ Open ☐ Transanal ☐ Laparoscopic

If symptoms persist after a "successful" pull-through, what is your management?

☐ Conservative with enemas and laxatives ☐ Botox injection

☐ Posterior myectomy ☐ Redo pull-through ☐ other *(please specify)* ....

In patients with Trisomy 21 and HD, do you change your surgical approach?

☐ Yes ☐ No

If Yes, in which way? ....

Total colonic aganglionosis (TCI)

When do you plan the pull-through?

☐ Neonatal period ☐ 1-6 months ☐ 6-12 months ☐ > 12 months ☐ older

In TCA, if you create a stoma, where do you site it?

☐ after intraoperative biopsies ☐ according to the radiological findings

☐ always in the ileum ☐ according to the optical impression during surgery

Which type of pull-through do you favour? ☐ Soave ☐ Swenson ☐ Duhamel

Would you perform a J pouch in a patient with TCI? ☐ Yes ☐ No

Please leave this form at registration desk.

Thanks for your time!

EUPSA survey (2017) adapted and translated into Portuguese

QUESTIONÁRIO BRASILEIRO SOBRE DOENÇA DE HIRSCHSPRUNG

Chefe de serviço/ Médico ou docente de cirurgia pediátrica / médico residente *(Por favor, circule se apropriado)*

Cidade de atuação/ Estado: ______________________________

Quantos casos de doença de Hirschsprung (DH) o hospital que você trabalha atende por ano?

( ) < 10 ( ) 10-20 ( ) > 20

Diagnóstico

Na avaliação de pacientes com suspeita de DH, quais testes você realiza? *(Pode assinalar mais de um)*

( ) Biópsia retal ( ) Enema contrastado ( ) Manometria anorretal

No caso de biopsia retal, qual a sua escolha?

( ) Biopsia de sucção ( ) Biopsia aberta

No caso de biopsia de sucção,

- **quantos fragmentos de biopsia você normalmente obtém?** ( ) 1 ( ) 2 ( ) 3 ( ) >3
- **a quantos cm da linha denteada é a sua biopsia mais distal?** ( ) 1 ( ) 2 ( ) 3 ( ) >3

Quais métodos de análise seu patologista usa para as biopsias retais? *(Pode assinalar mais de um)*

( ) Hematoxilina/eosina ( ) Acetilcolinesterase

( ) Calretinina ( ) Outro *(por favor especifique)* ....

Em quanto tempo você obtém os resultados de uma biopsia retal?

( ) <24 h ( ) 24-48 h ( ) 3-5 dias ( ) >5dias

Quais das seguintes entidades você reconhece a existência? *(Pode assinalar mais de uma)*

( ) Displasia neuronal intestinal do tipo B ( ) Hipoganglionose ( ) DH ultra curta

( ) *Desmosis coli* ( ) Nenhuma das anteriores ( ) Outra *(por favor especifique)* ....

Quais das seguintes entidades você já fez diagnóstico e tratou? *(Pode assinalar mais de uma)*

( ) Displasia neuronal intestinal do tipo B (DNI-B) ( ) Hipoganglionose ( ) DH ultra curta

( ) *Desmosis coli* ( ) Nenhuma das anteriores

( ) Outra *(por favor especifique)* ....

Já fez o diagnóstico de alguma destas entidades em associação à DH?

( ) Não ( ) Sim Se sim, qual (is)?...

Cirurgia

Em um recém nascido com DH confirmada, qual a sua conduta imediata?

( ) Cirurgia ao diagnóstico ( ) Cirurgia tardia

Se você opta pela cirurgia retardada, em qual idade você planeja realizar o abaixamento?..........

Enquanto espera pela cirurgia, como você mantém o cólon descomprimido?

( ) Irrigações retais em casa ( ) Dilatações/estímulos retais em casa

( ) Abaixamento primário logo após o diagnóstico ( ) Estoma

( ) Outro *(por favor especifique)* ....

Se você realiza um estoma em um paciente com DH típica em retosigmóide, qual o local escolhido?

( ) sigmóide ( ) cólon transverso direito ( ) íleo

( )ao nível da transição de dilatação do colon

Em que idade você planeja realizar o abaixamento?

( ) período neonatal ( )1-6 meses ( ) 6-12 meses ( ) >12 meses ( ) mais velho

Qual tipo de abaixamento é sua primeira escolha?

( ) Soave ( ) Swenson ( ) Duhamel

Qual sua abordagem preferencial para o abaixamento?

( ) Aberta ( ) Transanal ( ) Laparoscópica

Se os sintomas persistirem após um abaixamento adequado e correto, qual a sua conduta?

( ) Conservadora com enemas e laxantes ( ) Injeção de botox

( ) Miectomia posterior ( ) Reabaixamento ( ) Outro *(por favor especifique)* ....

Em pacientes com a Trissomia do 21 e DH, você muda o seu planejamento cirúrgico?

( ) Sim ( ) Não

Se sim, de que maneira?..

<u>Aganglionose colônica total (ACT)</u>

Quando você planeja o abaixamento?

( ) Período neonatal ( ) 1-6 meses ( ) 6-12 meses ( ) > 12 meses ( ) mais velho

Na ACT, se você for criar um estoma, onde você o localiza?

( ) decido após biopsias intraoperatória ( ) decido de acordo com os achados radiológicos

( ) sempre no íleo ( ) decido de acordo com a impressão macroscópica durante a cirurgia

Qual tipo de abaixamento você escolhe?

( ) Soave ( ) Swenson ( ) Duhamel ( ) Outro *(por favor especifique)* ....

Você faria uma bolsa em "J" em um paciente com ACT? ( ) Sim ( ) Não

Supplementary Material S4

Pre-Test: "Brazilian Questionnaire on Hirschsprung's Disease"

PRE TESTE - "QUESTIONÁRIO BRASILEIRO SOBRE DOENÇA DE HIRSCHSPRUNG"

TERMO DE CONSENTIMENTO LIVRE E ESCLARECIDO

Convido o Senhor (a) para participar do projeto de pesquisa intitulado "Manejo diagnóstico e terapêutico da Doença de Hirschsprung: um levantamento com cirurgiões pediátricos do Brasil", que é desenvolvido por mim, Cesar Saul Quevedo Penaloza, médico, com orientação do profissional Pedro Luiz Toledo de Arruda Lourenção, médico, cirurgião pediátrico e professor associado da Faculdade de Medicina de Botucatu – UNESP.

O objetivo deste estudo é a aplicação de questionário a cirurgiões pediátricos sobre o manejo da doença de Hirschsprung em crianças brasileiras, visando descrever e comparar as condutas diagnósticas e terapêuticas mais utilizadas no país com outros levantamentos já publicados em outros países.

Uma das etapas do projeto consiste na fase de pré-teste, na qual o questionário inicialmente elaborado será avaliado por 20 cirurgiões pediátricos em sua língua nativa. Convidamos o senhor (a) a ser um destes avaliadores. Informamos que a sua opinião será utilizada para confecção e validação da versão final do questionário.

Sua participação se dará através da leitura do questionário e avaliação de seu grau de entendimento, de acordo com uma escala numérica. Você também poderá emitir sugestões para a melhor redação e entendimento do questionário, sem alteração de seu conteúdo. Isto demandará aproximadamente 10 minutos. Os riscos relacionados à confidencialidade de sua participação estão minimizados pela garantia da confidencialidade de seus dados. Assim, caso concorde em participar deste estudo, seu nome não será divulgado e você não será identificado (a) em nenhuma publicação que possa existir a partir desse estudo. Fique ciente de que sua participação neste estudo é voluntária e que mesmo após ter dado seu consentimento para participar da pesquisa, você poderá retirá-lo a qualquer momento.

Este termo será emitido em duas vias, sendo que uma via ficará sob responsabilidade do pesquisador e a outra via será fornecida a você, a qual recomendamos que seja guardada em seus arquivos. A participação no estudo não acarretará custos para você e não será disponível nenhuma compensação financeira adicional.

***Em caso de dúvidas com respeito aos aspectos éticos deste estudo, você poderá consultar:*

CEP- Comitê de Ética em Pesquisa da Faculdade de Medicina de Botucatu - UNESP
Chácara Butignoli s/n, Rubião Júnior - Botucatu - São Paulo - CEP: 18618-970
Telefones: (14) 3880-1608/3880-1609

Pesquisador Responsável: Pedro Luiz Toledo de Arruda Lourenção
Departamento de Cirurgia e Ortopedia da Faculdade de Medicina de Botucatu - UNESP
Avenida Prof. Montenegro, Distrito de Rubião Júnior, s/n - Botucatu - São Paulo – CEP 18.618-970
Telefones: (14) 3880- 1703
E-mail: pedro.lourencao@.unesp.br

Aluno de mestrado: Cesar Saul Quevedo Penaloza
Orientador: Pedro Luiz Toledo de Arruda Lourenção

CERTIFICADO DE CONSENTIMENTO

Eu,____, fui informado (a) dos objetivos da pesquisa acima de maneira clara e detalhada e esclareci minhas dúvidas. Sei que em qualquer momento poderei solicitar novas informações e modificar minha decisão se assim o desejar. Declaro que CONCORDO em participar de forma voluntária, estando ciente que todos os meus dados estarão resguardos, através do sigilo que os pesquisadores se comprometeram. Estou ciente que os resultados desse estudo poderão ser publicados em revistas científicas sem, no entanto, que minha identidade seja revelada. Recebi uma cópia deste termo de consentimento livre e esclarecido e me foi dada a oportunidade de ler e esclarecer as minhas dúvidas.

____

Nome Assinatura do Participante Data

____

Nome Assinatura do Pesquisador Data

ORIENTAÇÕES

A seguir serão apresentadas as perguntas do questionário proposto.

Solicitamos que o(a) senhor(a) leia as perguntas e avalie o seu entendimento sobre as perguntas com eventuais sugestões.

1. Atualmente, você exerce o cargo de:
a. Chefe de serviço b. Médico ou docente da Cirurgia Pediátrica c. Médico residente

Em relação à pergunta anterior, em uma Escala Verbal Numérica (EVN) de 1 (não entendi nada) a 5 (entendi perfeitamente e não tenho dúvidas), classifique seu grau de entendimento.

1- Não entendi nada	2	3	4	5- Entendi perfeitamente e não tenho dúvidas
O	O	O	O	O

Alguma sugestão para melhor redação?
___

2. Em que cidade/estado do Brasil trabalha?

Em relação à pergunta anterior, em uma Escala Verbal Numérica (EVN) de 1 (não entendi nada) a 5 (entendi perfeitamente e não tenho dúvidas), classifique seu grau de entendimento.

1- Não entendi nada	2	3	4	5- Entendi perfeitamente e não tenho dúvidas
O	O	O	O	O

Alguma sugestão para melhor redação?
___

3. Quantos pacientes com Doença de Hirschsprung (DH) são atendidos por ano no hospital onde você trabalha?
a. <10 b. 10-20 c. >20

Em relação à pergunta anterior, em uma Escala Verbal Numérica (EVN) de 1 (não entendi nada) a 5 (entendi perfeitamente e não tenho dúvidas), classifique seu grau de entendimento.

1- Não entendi nada	2	3	4	5- Entendi perfeitamente e não tenho dúvidas
O	O	O	O	O

Alguma sugestão para melhor redação?
___

4. Quantos destes são casos novos?

Em relação à pergunta anterior, em uma Escala Verbal Numérica (EVN) de 1 (não entendi nada) a 5 (entendi perfeitamente e não tenho dúvidas), classifique seu grau de entendimento.

1- Não entendi nada	2	3	4	5- Entendi perfeitamente e não tenho dúvidas
O	O	O	O	O

Alguma sugestão para melhor redação?
___

Aluno de mestrado: Cesar Saul Quevedo Penaloza
Orientador: Pedro Luiz Toledo de Arruda Lourenção

5. Qual é o método diagnóstico que define a sua conduta? (Assinalar só uma)
a. Biópsia retal b. Enema contrastado c. Manometria anorretal

Em relação à pergunta anterior, em uma Escala Verbal Numérica (EVN) de 1 (não entendi nada) a 5 (entendi perfeitamente e não tenho dúvidas), classifique seu grau de entendimento.

1- Não entendi nada	2	3	4	5- Entendi perfeitamente e não tenho dúvidas
O	O	O	O	O

Alguma sugestão para melhor redação?
____

6. Na avaliação de pacientes com suspeita de DH, quais testes você realiza? (Pode assinalar mais de um)
a. Biópsia retal b. Enema contrastado c. Manometria anorretal

Em relação à pergunta anterior, em uma Escala Verbal Numérica (EVN) de 1 (não entendi nada) a 5 (entendi perfeitamente e não tenho dúvidas), classifique seu grau de entendimento.

1- Não entendi nada	2	3	4	5- Entendi perfeitamente e não tenho dúvidas
O	O	O	O	O

Alguma sugestão para melhor redação?
____

7. No caso de biópsia retal, qual é sua escolha?
a. Biópsia de sucção
b. Biópsia aberta / Cirúrgica (Técnica de Swenson)
c. Outra (por favor especifique) ____________________

Em relação à pergunta anterior, em uma Escala Verbal Numérica (EVN) de 1 (não entendi nada) a 5 (entendi perfeitamente e não tenho dúvidas), classifique seu grau de entendimento.

1- Não entendi nada	2	3	4	5- Entendi perfeitamente e não tenho dúvidas
O	O	O	O	O

Alguma sugestão para melhor redação?
____

8. No caso de biópsia de sucção, quantos fragmentos de biópsia você normalmente obtém?
a. 1 fragmento b. 2 fragmentos c. 3 fragmentos d. >3 fragmentos

Em relação à pergunta anterior, em uma Escala Verbal Numérica (EVN) de 1 (não entendi nada) a 5 (entendi perfeitamente e não tenho dúvidas), classifique seu grau de entendimento.

1- Não entendi nada	2	3	4	5- Entendi perfeitamente e não tenho dúvidas
O	O	O	O	O

Alguma sugestão para melhor redação?
____

Aluno de mestrado: Cesar Saul Quevedo Penaloza
Orientador: Pedro Luiz Toledo de Arruda Lourenção

9. No caso de biópsia de sucção, a quantos centímetros (cm) da linha denteada é a sua biópsia mais distal?
a. 1 cm b. 2 cm c. 3 cm d. >3 cm

Em relação à pergunta anterior, em uma Escala Verbal Numérica (EVN) de 1 (não entendi nada) a 5 (entendi perfeitamente e não tenho dúvidas), classifique seu grau de entendimento.

1- Não entendi nada	2	3	4	5- Entendi perfeitamente e não tenho dúvidas
O	O	O	O	O

Alguma sugestão para melhor redação?
____

10. Quais métodos de análise seu patologista usa para as biópsias retais? (Pode assinalar mais de um)
a. Hematoxilina/eosina b. Acetilcolinesterase c. Calretinina
d. Outro (por favor especifique) _____________

Em relação à pergunta anterior, em uma Escala Verbal Numérica (EVN) de 1 (não entendi nada) a 5 (entendi perfeitamente e não tenho dúvidas), classifique seu grau de entendimento.

1- Não entendi nada	2	3	4	5- Entendi perfeitamente e não tenho dúvidas
O	O	O	O	O

Alguma sugestão para melhor redação?
____

11. Em quanto tempo você obtém os resultados de uma biópsia retal?
a. < 24 horas b. 24-48 horas c. 3-5 dias d. >5 dias

Em relação à pergunta anterior, em uma Escala Verbal Numérica (EVN) de 1 (não entendi nada) a 5 (entendi perfeitamente e não tenho dúvidas), classifique seu grau de entendimento.

1- Não entendi nada	2	3	4	5- Entendi perfeitamente e não tenho dúvidas
O	O	O	O	O

Alguma sugestão para melhor redação?
____

12. Você não realiza biópsia retal por que?
a. Não está disponível no meu serviço
b. Penso ser dispensável se manometria ou enema opaco forem positivos para DH
c. Outro (por favor especifique) ____________________

Em relação à pergunta anterior, em uma Escala Verbal Numérica (EVN) de 1 (não entendi nada) a 5 (entendi perfeitamente e não tenho dúvidas), classifique seu grau de entendimento.

1- Não entendi nada	2	3	4	5- Entendi perfeitamente e não tenho dúvidas
O	O	O	O	O

Alguma sugestão para melhor redação?
____

Aluno de mestrado: Cesar Saul Quevedo Penaloza
Orientador: Pedro Luiz Toledo de Arruda Lourenção

13. Quais das seguintes entidades você reconhece a existência? (Pode assinalar mais de uma)
a. Displasia neuronal intestinal do tipo B (DNI-B) **b. Hipoganglionose**
c. DH ultra curta **d. *Desmosis coli.*** **e. Nenhuma das anteriores**
f. Outra (por favor especifique) ____________________

Em relação à pergunta anterior, em uma Escala Verbal Numérica (EVN) de 1 (não entendi nada) a 5 (entendi perfeitamente e não tenho dúvidas), classifique seu grau de entendimento.

1- Não entendi nada	2	3	4	5- Entendi perfeitamente e não tenho dúvidas
O	O	O	O	O

Alguma sugestão para melhor redação?
____

14. Quais das seguintes entidades você já fez diagnóstico e tratou? (Pode assinalar mais de uma)
a. Displasia neuronal intestinal do tipo B (DNI-B) **b. Hipoganglionose**
c. DH ultra curta **d. *Desmosis coli.*** **e. Nenhuma das anteriores**
f. Outra (por favor especifique) ____________________

Em relação à pergunta anterior, em uma Escala Verbal Numérica (EVN) de 1 (não entendi nada) a 5 (entendi perfeitamente e não tenho dúvidas), classifique seu grau de entendimento.

1- Não entendi nada	2	3	4	5- Entendi perfeitamente e não tenho dúvidas
O	O	O	O	O

Alguma sugestão para melhor redação?
____

15. Já fez o diagnóstico de alguma destas entidades em associação à DH?
a. Não **b. Sim (por favor especifique qual (is)?)** ____________________

Em relação à pergunta anterior, em uma Escala Verbal Numérica (EVN) de 1 (não entendi nada) a 5 (entendi perfeitamente e não tenho dúvidas), classifique seu grau de entendimento.

1- Não entendi nada	2	3	4	5- Entendi perfeitamente e não tenho dúvidas
O	O	O	O	O

Alguma sugestão para melhor redação?
____

16. Em um recém-nascido com DH confirmada, qual é a sua conduta imediata?
a. Abaixamento do cólon logo após diagnóstico **b. Postergar abaixamento do cólon**

Em relação à pergunta anterior, em uma Escala Verbal Numérica (EVN) de 1 (não entendi nada) a 5 (entendi perfeitamente e não tenho dúvidas), classifique seu grau de entendimento.

1- Não entendi nada	2	3	4	5- Entendi perfeitamente e não tenho dúvidas
O	O	O	O	O

Alguma sugestão para melhor redação?
____

17. Se você optar por postergar o abaixamento do cólon, quando você planeja realizá-lo?
a. Período neonatal b. 1-6 meses c. 6-12 meses d. 1-2 anos
e. 2-4 anos f. >4 anos g. Nunca adio a realização do abaixamento de cólon

Em relação à pergunta anterior, em uma Escala Verbal Numérica (EVN) de 1 (não entendi nada) a 5 (entendi perfeitamente e não tenho dúvidas), classifique seu grau de entendimento.

1- Não entendi nada	2	3	4	5- Entendi perfeitamente e não tenho dúvidas
O	O	O	O	O

Alguma sugestão para melhor redação?

____

18. Enquanto espera pela cirurgia, como você mantém o cólon descomprimido? (Pode assinalar mais de uma)
a. Irrigações retais em casa b. Dilatações/estímulos retais em casa
c. Estoma d. Outra (por favor especifique) ____________________

Em relação à pergunta anterior, em uma Escala Verbal Numérica (EVN) de 1 (não entendi nada) a 5 (entendi perfeitamente e não tenho dúvidas), classifique seu grau de entendimento.

1- Não entendi nada	2	3	4	5- Entendi perfeitamente e não tenho dúvidas
O	O	O	O	O

Alguma sugestão para melhor redação?

____

19. Quando é necessário, qual local você escolhe para realizar um estoma em um paciente com DH de forma clássica (em retossigmóide)?
a. Sigmoide b. Cólon transverso direito c. Íleo
d. Ao nível da transição de dilatação do cólon e. Outra (por favor especifique) __________

Em relação à pergunta anterior, em uma Escala Verbal Numérica (EVN) de 1 (não entendi nada) a 5 (entendi perfeitamente e não tenho dúvidas), classifique seu grau de entendimento.

1- Não entendi nada	2	3	4	5- Entendi perfeitamente e não tenho dúvidas
O	O	O	O	O

Alguma sugestão para melhor redação?

____

20. Qual tipo de abaixamento é sua primeira escolha? (Técnica para abaixamento)
a. Soave b. Swenson c. Duhamel

Em relação à pergunta anterior, em uma Escala Verbal Numérica (EVN) de 1 (não entendi nada) a 5 (entendi perfeitamente e não tenho dúvidas), classifique seu grau de entendimento.

1- Não entendi nada	2	3	4	5- Entendi perfeitamente e não tenho dúvidas
O	O	O	O	O

Alguma sugestão para melhor redação?

____

Aluno de mestrado: Cesar Saul Quevedo Penaloza
Orientador: Pedro Luiz Toledo de Arruda Lourenção

21. Qual sua abordagem preferencial para o abaixamento? (Via para abaixamento)
a. Aberta b. Transanal c. Laparoscópica

Em relação à pergunta anterior, em uma Escala Verbal Numérica (EVN) de 1 (não entendi nada) a 5 (entendi perfeitamente e não tenho dúvidas), classifique seu grau de entendimento.

1- Não entendi nada	2	3	4	5- Entendi perfeitamente e não tenho dúvidas
O	O	O	O	O

Alguma sugestão para melhor redação?
____

22. Se os sintomas persistirem após um abaixamento adequado e correto, qual a sua conduta?
a. Conservadora com enemas e laxantes b. Injeção de botox
c. Miectomia posterior d. Reabaixamento e. Outra (por favor especifique) _____

Em relação à pergunta anterior, em uma Escala Verbal Numérica (EVN) de 1 (não entendi nada) a 5 (entendi perfeitamente e não tenho dúvidas), classifique seu grau de entendimento.

1- Não entendi nada	2	3	4	5- Entendi perfeitamente e não tenho dúvidas
O	O	O	O	O

Alguma sugestão para melhor redação?
____

23. Em pacientes com Trissomia do 21 e DH, você muda o seu planejamento cirúrgico?
a. Não b. Sim, de que maneira? (por favor especifique) ____________________

Em relação à pergunta anterior, em uma Escala Verbal Numérica (EVN) de 1 (não entendi nada) a 5 (entendi perfeitamente e não tenho dúvidas), classifique seu grau de entendimento.

1- Não entendi nada	2	3	4	5- Entendi perfeitamente e não tenho dúvidas
O	O	O	O	O

Alguma sugestão para melhor redação?
____

24. Em um paciente com Aganglionose colônica total (ACT), quando você planeja o abaixamento?
a. Período neonatal b. 1-6 meses c. 6-12 meses
d. 1-2 anos e. 2-4 anos f. >4 anos

Em relação à pergunta anterior, em uma Escala Verbal Numérica (EVN) de 1 (não entendi nada) a 5 (entendi perfeitamente e não tenho dúvidas), classifique seu grau de entendimento.

1- Não entendi nada	2	3	4	5- Entendi perfeitamente e não tenho dúvidas
O	O	O	O	O

Alguma sugestão para melhor redação?
____

Aluno de mestrado: Cesar Saul Quevedo Penaloza
Orientador: Pedro Luiz Toledo de Arruda Lourenção

25. Na ACT, se você for criar um estoma, onde você o localiza?
a. Decido após biópsias intraoperatória
b. Decido de acordo com os achados radiológicos
c. Sempre no íleo
d. Decido de acordo com a impressão macroscópica durante a cirurgia

Em relação à pergunta anterior, em uma Escala Verbal Numérica (EVN) de 1 (não entendi nada) a 5 (entendi perfeitamente e não tenho dúvidas), classifique seu grau de entendimento.

1- Não entendi nada	2	3	4	5- Entendi perfeitamente e não tenho dúvidas
O	O	O	O	O

Alguma sugestão para melhor redação?

____

26. Na ACT, qual tipo de abaixamento você escolhe? (Tipo de técnica)
a. Soave b. Swenson c. Duhamel / Martin d. Outra (por favor especifique)

Em relação à pergunta anterior, em uma Escala Verbal Numérica (EVN) de 1 (não entendi nada) a 5 (entendi perfeitamente e não tenho dúvidas), classifique seu grau de entendimento.

1- Não entendi nada	2	3	4	5- Entendi perfeitamente e não tenho dúvidas
O	O	O	O	O

Alguma sugestão para melhor redação?

____

27. Você faria uma bolsa em "J" em um paciente com ACT?
a. Sim b. Não

Em relação à pergunta anterior, em uma Escala Verbal Numérica (EVN) de 1 (não entendi nada) a 5 (entendi perfeitamente e não tenho dúvidas), classifique seu grau de entendimento.

1- Não entendi nada	2	3	4	5- Entendi perfeitamente e não tenho dúvidas
O	O	O	O	O

Alguma sugestão para melhor redação?

____

Fim do questionário.

Supplementary Material S5

Final version: "Brazilian Questionnaire on Hirschsprung's Disease"

TERMO DE CONSENTIMENTO LIVRE E ESCLARECIDO

Convido o Senhor(a) para participar do projeto de pesquisa intitulado **"Manejo diagnóstico e terapêutico da Doença de Hirschsprung: um levantamento com cirurgiões pediátricos do Brasil"**, que é desenvolvido por mim, Cesar Saul Quevedo Penaloza, médico residente da cirurgia do aparelho digestivo da UNESP, sob orientação do Dr. Pedro Luiz Toledo de Arruda Lourenção, médico, cirurgião pediátrico e professor associado da Faculdade de Medicina de Botucatu – UNESP. O objetivo deste estudo é a aplicação de questionário a cirurgiões pediátricos sobre o manejo da doença de Hirschsprung em crianças brasileiras, visando descrever e comparar as condutas diagnósticas e terapêuticas mais utilizadas no país com outros levantamentos já publicados em outros países. Sua participação se dará através da resposta ao questionário. Isto demandará aproximadamente 5 minutos. Os riscos relacionados à confidencialidade de sua participação estão minimizados. Caso concorde em participar deste estudo, seu nome não será divulgado e você não será identificado(a) em nenhuma publicação que possa existir a partir desse estudo. Fique ciente de que sua participação neste estudo é voluntária e que mesmo após ter dado seu consentimento para participar da pesquisa, você poderá retirá-lo a qualquer momento. A participação no estudo não acarretará custos para você e não será disponível nenhuma compensação financeira adicional.

○ Concorda e aceita participar no estudo
○ Discorda e por isso não aceita participar do estudo

Em caso de dúvidas com respeito aos aspectos éticos deste estudo, você poderá consultar:
CEP - Comitê de Ética em Pesquisa da Faculdade de Medicina de Botucatu – UNESP
Chácara Butignoli s/n, Rubião Júnior - Botucatu - São Paulo - CEP: 18618-970
Telefones: (14) 3880-1608/3880-1609
Pesquisador Responsável: Pedro Luiz Toledo de Arruda Lourenção
Departamento de Cirurgia e Ortopedia da Faculdade de Medicina de Botucatu – UNESP
Avenida Prof. Montenegro, Distrito de Rubião Júnior, s/n - Botucatu - São Paulo - CEP 18.618-970
Contato: hirschsprung.fmb@unesp.br

______________________________ Assinatura do participante

______________________________ Dr Cesar Saul Quevedo Penaloza
Autor do projeto

Manejo diagnóstico e terapêutico da Doença de Hirschsprung: um levantamento com cirurgiões pediátricos do Brasil

QUESTIONÁRIO

1. Identificação (nome completo do participante)

____

A identificação é solicitada apenas para controle do número de participantes do estudo. Ela não será vinculada às respostas do seu questionário e não haverá divulgação dos nomes em nenhuma publicação que possa existir a partir desse estudo.

2. No serviço de cirurgia pediátrica, onde você mais trabalha atualmente, você exerce o cargo de:

○ Chefe de serviço ○ Médico ou docente ○ Médico residente

3. O serviço de cirurgia pediátrica, onde você mais trabalha atualmente, está localizado em que cidade/estado do Brasil?

____

4. No serviço de cirurgia pediátrica, onde você mais trabalha atualmente, quantos pacientes com Doença de Hirschsprung são atendidos por ano?

○ < 10 ○ 10 – 20 ○ > 20

5. No serviço de cirurgia pediátrica, onde você mais trabalha atualmente, quantos casos novos de pacientes com DH são atendidos por ano? *DH: Doença de Hirschsprung*

____

6. Na avaliação de pacientes com suspeita de DH, qual é o método que você usa para definir o diagnóstico?

○ Biópsia retal ○ Enema contrastado ○ Manometria anorretal

7. Na avaliação de pacientes com suspeita de DH, quais testes você realiza? *(Pode assinalar mais de um)*

○ Biópsia retal ○ Enema contrastado ○ Manometria anorretal

○ Outro (por favor especifique): ______________________________________

Se na pergunta 7:
- "foi assinalada Biopsia Retal", responda as perguntas 8 a 13, e continue na pergunta 15.
- "não foi assinalada Biopsia Retal", responda a pergunta 14 e continue na pergunta 15.

8. No caso de biópsia retal, em um recém-nascido ou lactente, qual técnica você mais realiza?

○ Biópsia de sucção

○ Biópsia aberta / Cirúrgica (Técnica de Swenson)

○ Outro (por favor especifique): ______________________________________

9. No caso de biópsia retal, em uma criança com mais de 3 anos de idade, qual técnica você mais realiza?

○ Biópsia de sucção

○ Biópsia aberta / Cirúrgica (Técnica de Swenson)

○ Outro (por favor especifique): ____________________

10. Se você optar por biópsia de sucção, quantos fragmentos de biópsia você normalmente obtém?

○ 1 fragmento ○ 2 fragmentos ○ 3 fragmentos ○ >3 fragmentos (por favor quantifique)

11. Se você optar por biópsia de sucção, a quantos centímetros(cm) da linha denteada é a sua biópsia mais distal?

○ 1 cm ○ 2 cm ○ 3 cm ○ >3 cm

12. Quais métodos de análise seu patologista usa para as biópsias retais? *(Pode assinalar mais de um)*

○ Hematoxilina/eosina ○ Acetilcolinesterase ○ Calretinina

○ Outro (por favor especifique): ____________________

13. Em quanto tempo você obtém os resultados de uma biópsia retal?

○ < 24 horas ○ 24-48 horas ○ 3-5 dias

○ >5 dias (por favor especifique)____________________

14. Por qual motivo você "não" realiza biópsia retal?

○ Não está disponível no meu serviço

○ Penso ser dispensável se manometria ou enema opaco forem positivos para DH

○ Outro (por favor especifique): ____________________

15. Quais das seguintes entidades você reconhece a existência? *(Pode assinalar mais de uma)*

○ Displasia neuronal intestinal do tipo B (DNI-B) ○ Hipoganglionose

○ DH ultra curta ○ *Desmosis coli* ○ Nenhuma das anteriores

○ Outra (por favor especifique): ____________________

16. Quais das seguintes entidades você já fez diagnóstico e tratou? *(Pode assinalar mais de uma)*

○ Displasia neuronal intestinal do tipo B (DNI-B) ○ Hipoganglionose

○ DH ultra curta ○ *Desmosis coli* ○ Nenhuma das anteriores

○ Outra (por favor especifique): ____________________

17. Já fez o diagnóstico de alguma destas entidades em associação à DH?

○ Não

○ Sim (por favor especifique quais)____________________

18. Em um recém-nascido com DH confirmada e estável clinicamente, qual é a sua conduta imediata?

○ Abaixamento do cólon logo após diagnóstico ○ Postergar abaixamento do cólon

19. Em um recém-nascido com DH confirmada e estável clinicamente, se você optar por postergar o abaixamento do cólon, quando você planeja realizá-lo?

○ Período neonatal ○ 1-6 meses ○ 6-12 meses ○ 1-2 anos ○ 2-4 anos ○ >4 anos

○ Nunca adio a realização do abaixamento de cólon

20. Em um recém-nascido com DH confirmada e estável clinicamente, enquanto espera pela cirurgia, como você mantém o cólon descomprimido? *(Pode assinalar mais de uma)*

○ Irrigações retais em casa ○ Dilatações/estímulos retais em casa ○ Estoma

○ Outra (por favor especifique): ____

21. Quando é necessário, qual local você escolhe para realizar um estoma em um paciente com DH de forma clássica (em retossigmoide)?

○ Sigmoide ○ Cólon transverso direito ○ Íleo ○ Ao nível da transição de dilatação do cólon

○ Outro (por favor especifique): ____

22. Em um recém-nascido ou lactente com DH confirmada, qual tipo de abaixamento é sua primeira escolha? *(Técnica para abaixamento)*

○ Soave ou De La Torre - Mondragon (via transanal) ○ Swenson (via abdominoperineal ou transanal)

○ Duhamel

23. Em um recém-nascido ou lactente com DH confirmada, qual seria sua abordagem preferencial para o abaixamento? *(Via para abaixamento)*

○ Aberta ○ Transanal ○ Laparoscópica

24. Em uma criança com mais de 3 anos de idade com DH confirmada, qual tipo de abaixamento é sua primeira escolha? *(Técnica para abaixamento)*

○ Soave ou De La Torre - Mondragon (via transanal) ○ Swenson (via abdominoperineal ou transanal)

○ Duhamel

25. Em uma criança com mais de 3 anos de idade com DH confirmada, qual seria sua abordagem preferencial para o abaixamento? *(Via para abaixamento)*

○ Aberta ○ Transanal ○ Laparoscópica

26. Se os sintomas persistirem após um abaixamento adequado e correto, qual a sua conduta?

○ Conservadora com enemas e laxantes ○ Injeção de botox

○ Miectomia posterior ○ Reabaixamento

○ Outro (por favor especifique): ____

27. Em pacientes com Trissomia do 21 e DH, você muda o seu planejamento cirúrgico?

○ Não

○ Sim, de que maneira? (por favor especifique) ________________________________

28. Em um paciente com aganglionose colônica total, quando você planeja o abaixamento?

○ Período neonatal ○ 1-6 meses ○ 6-12 meses ○ 1-2 anos ○ 2-4 anos

○ >4 anos (por favor especifique)____

29. Em um paciente com aganglionose colônica total, se você for criar um estoma, onde você o localiza?

○ Decido após biópsias intraoperatória

○ Decido de acordo com os achados radiológicos

○ Sempre no íleo

○ Decido de acordo com a impressão macroscópica durante a cirurgia

30. Em um paciente com Aganglionose colônica total, qual tipo de abaixamento você escolhe? *(Tipo de técnica)*

○ Soave ou De La Torre - Mondragon (via transanal)

○ Swenson (via abdominoperineal ou transanal)

○ Duhamel / Martin

○ Outro (por favor especifique): ____

31. Em um paciente com Aganglionose colônica total, você faria uma bolsa ileal em "J"?

○ Sim ○ Não

Apoio:

FMB Faculdade de Medicina Botucatu 1963

Associação Brasileira de Cirurgia Pediátrica Fundada em 30 de janeiro de 1964

CIPESP

FAPESP

Processo nº 2021/02189-0
